# Efficacy of Chuanxiong Ding Tong Herbal Formula Granule in the Treatment and Prophylactic of Migraine Patients: A Randomized, Double-Blind, Multicenter, Placebo-Controlled Trial

**DOI:** 10.1155/2012/967968

**Published:** 2012-11-29

**Authors:** Caihong Fu, Lihua Yu, Yihuai Zou, Kegang Cao, Jianjun Zhao, Haiyang Gong, Shuquan Zhang, Anji Lin, Mengjiu Dong, Wenming Yang, Tao Li, Liyun He, Fei Su, Ruolan Wu, Dongdong Lin

**Affiliations:** ^1^Department of Neurology, Dongzhimen Hospital, Beijing University of Chinese Medicine, No. 5 Haiyun Cang, Dong Cheng District, Beijing 100700, China; ^2^Key Laboratory for Internal Chinese Medicine of Ministry of Education and Beijing, Beijing University of Traditional Chinese Medicine, No. 5 Haiyun Cang, Dong Cheng District, Beijing 100700, China; ^3^Department of Neurology, The Affiliated Hospital to Changchun University of Chinese Medicine, No. 1478 Gongnong Avenue, Chaoyang District, Changchun 130021, China; ^4^Department of Traditional Chinese Medicine, Beijing Tiantan Hospital, Capital Medical University, No. 6 Tiantan Xili, Dong Cheng District, Beijing 100050, China; ^5^Department of Neurology, Taian Hospital of Traditional Chinese Medicine, No. 216 Yingxuan Avenue, Taian 271000, China; ^6^Department of Neurology, Xiamen Hospital of Traditional Chinese Medicine, No. 1739 Xianyue Road, Xiamen 361001, China; ^7^Department of Neurology, Hubei Hospital of Traditional Chinese Medicine, No. 4 Huayuan Shan,Wuchang District, Wuhan 430061, China; ^8^Department of Neurology, The First Subsidiary Affiliated Hospital of Anhui College of Traditional Chinese Medicine, No.117 Meishan Road, Hefei 230031, China; ^9^Department of Neurology, Xiyuan Hospital, China Academy of Chinese Medical Sciences, No. 1 Xiyuan Playground, Haidian District, Beijing 100091, China; ^10^China Academy of Chinese Medical Sciences, No. 16 South Street, Dong zhimen nei, Dong Cheng District, Beijing 100700, China

## Abstract

*Objective. *To evaluate the efficacy of traditional Chinese herbal ChuanXiong Ding Tong herbal formula granule (CXDT-HFG) for migraine patients with “the Syndrome of Liver Wind and Blood Stasis.” *Methods. *150 migraine patients were recruited and assigned randomly in a double-blind, placebo-controlled study to receive CXDT-HFG (*n* = 99) plus necessary analgesics, or placebo (*n* = 51) plus necessary analgesics for 16 weeks (12 weeks' intervention and 4 weeks' follow up). Outcome measures included migraine days, frequency of migraine attacks, analgesics consumption for acute treatment, and the proportion of responders as well as the visual analogue scale (VAS) scores and intensity for pain. *Results.* Compared with the placebo group, the CXDT-HFG group showed significant reduction in migraine days and attacks frequency at week 12 and follow-up period (*P* < 0.05) as well as in the reduction of VAS scores at follow-up period.There was significant difference in the proportion of responders between the two groups at follow-up period (*P* = 0.014). However there were no significant differences between the two groups in analgesics consumption (*P* > 0.05)*. Conclusion. *CXDT-HFG was more effective than placebo in decreasing days of migraine attacks, frequency, VAS scores, and relieving pain intensity for migraine patients.

## 1. Introduction

Migraine, a common and chronic headache, which is understood to be a neurovascular dysfunction, is characterized with recurrent headache attacks. The pain is located unilaterally, moderate to severe intensity, usually aggravated by physical activity, and usually lasts 4–72 hours. In addition, migraine attacks are often accompanied by nausea and/or vomiting, photophobia, phonophobia, etc. Migraine is high morbidity, procrastinating, and refractory, severely affecting the ability to work and quality of life [1–5]. The epidemiological study from the United States shows that the lifelong cumulative inDcidence of migraine is 7.4% in males and 21% in females [6]. The temporary functional disability, accompanying migraine [6], brings huge economic losses to the society. Migraine has been listed as one of the most serious, chronic, and dysfunctional diseases, which is equal to quadriplegia, mental disorders, and dementia by World Health Organization [7]. Due to its high incidence and high economic cost [2], the treatment and prevention of migraine has already attracted broad attention in the world in recent years.

Migraine is divided into the period of acute episode and chronic remission [8] and its treatment and prevention requires standard drug therapy and regular management [9–12]. There are many drugs used to treat acute migraine, such as aspirin, acetaminophen, nonsteroidal, anti-inflammatory drugs (NSAIDs), dihydroergotamine, and the triptans. Meanwhile Beta-blockers, calcium channel blockers, tricyclic antidepressant, and antiepileptic drugs have been used for migraine prevention [4, 13, 14]. However, in clinical practice it has been shown that these drugs can lead to headache or increase the frequency of migraine attacks and other side effects [15, 16], and which, to some extent, limit their application to migraine sufferers.

In the United States, Chinese herbal medicine, acupuncture and other natural therapy, have been licensed for use, and it is recognised clinical practice for herbal medicine to hold a place of importance in remedying headaches effectively and safely [17]. Clinical studies show that traditional acupuncture therapy can effectively reduce the migraine days and acute pain drug consumption, especially for migraine prevention, but no statistical differences are seen in the improvement of pain intensity [18, 19], thus sufferers still need drug treatment in the treatment and prevention of migraine. However, those studies on using Chinese herbal medicine to treat migraine with the method of multicenter, prospective, randomized, placebo controlled, double blind, are relatively few. According to the Guidelines for controlled trials of drugs in migraine (second edition) [20], this study chose migraine patients with “the Syndrome of Liver Wind and Blood Stasis” commonly seen in traditional Chinese medicine (TCM) clinic, and Chuanxiong Ding Tong herbal formula granule (CXDT-HFG) as treatment drug, so as to further evaluate the efficacy of Chinese herbal medicine in the treatment and prevention of migraine.

## 2. Materials and Methods

### 2.1. Study Design

**Figure 1:**
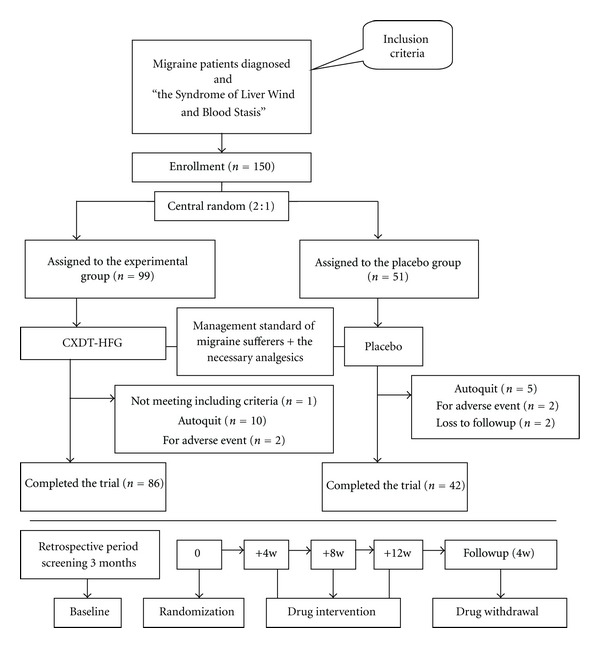
Flow diagram of the study progress about enrollment, randomization, intervention, and completion of the trial.

A multicenter, prospective, central-randomized, double-blind, placebo-controlled trial was conducted in this study. Migraine patients who met the inclusion criteria were randomly assigned into the experimental group and control group in a 2 : 1 ratio. The experimental group was treated with Chinese herbal medicine and the placebo group with placebo. Both groups were permitted to use the necessary analgesics during migraine acute attacks, as well as accepted regular management. The retrospective historical record of migraine attacks for nearly 3 months was defined as the baseline of the study. The study spans 16 weeks, including a treatment period of 12 weeks, and a follow-up period of 4 weeks. Headache diary was given to patients to record the details (migraine days, frequency, visual analogue scale (VAS) scores and acute medication, etc.) of migraine attacks during the trial period. These outcome measures such as migraine days, frequency of migraine attacks, VAS scores and intensity for pain were evaluated at 4 weeks, 8 weeks, 12 weeks, and follow-up period Figure1.

This study was designed and carried out cooperatively by methodologists and statisticians of the China Academy of Chinese Medical Sciences in Beijing. And the study protocol conforms to the Helsinki Declaration [21] and the research regulations for Chinese clinical trials. The Ethics Committee of the Affiliated Dongzhimen Hospital of Beijing University of Chinese Medicine reviewed and approved the study protocol. All participants signed informed consent before enrolment. 

### 2.2. Setting and Participants

150 migraine patients with “the Syndrome of Liver Wind and Blood Stasis” were recruited between January 2008 and June 2011 from outpatient departments in the following 8 hospitals: Dongzhimen Hospital affiliated to the Beijing University of Traditional Chinese Medicine, the Affiliated Hospital to Changchun University of Chinese Medicine, Beijing Tiantan Hospita affiliated to the Capital Medical University, Tai an Hospital of Traditional Chinese Medicine, Xiamen Hospital of Traditional Chinese Medicine, Hubei Hospital of Traditional Chinese Medicine, The First Subsidiary Hospital of Anhui College of Traditional Chinese Medicine, Xiyuan Hospital affiliated to the China Academy of Chinese Medical Sciences. All patients, with diagnosis of migraine with aura or without aura in accordance with the 2nd edition of the International Headache Society's International Classification of Headache Disorders (ICHD-II, IHS 2004) [8]. The study had a previous 3 months of retrospective record of migraine attacks (referred to as baseline) and 12-week double-blind, parallel group, placebo-controlled phase with trial drug treatment at day “0” of the double-blind phase, follow-up visit for 4 weeks after the experimental drug withdrawn (details will be described separately).

### 2.3. Diagnostic Criteria

The diagnostic criteria for this study were as follows. (1) Diagnosis standard in Western medicine: migraine without aura (MO) or with a typical aura (MA) as defined by 2004 HIS [8]. (2) TCM Differentiation standard: in accordance with the guiding principles for the clinical study of new drugs for use in traditional Chinese medicine released in 2002, combined with the characteristics of migraine, the standard of “Liver Wind and Blood Stasis syndrome” as follows: headache in the left or right, repeated attacks, severe pain, lasting for several hours or days, accompanyed by nausea, vomiting, vertigo, etc.; dark red or dark purple tongue, or tongue has bruises, ecchymosis or stasis points, thin-white fur, wiry pulse.

### 2.4. Inclusion Criteria

The inclusion criteria for this study were as follows: (1) diagnosed as migraine without aura (MO) or with a typical aura (MA) according to the diagnostic criteria specified by the International Classification of Headache Disorders; (2) meet the diagnostic standard of “Liver Wind and Blood Stasis syndrome” in TCM; (3) age of first onset ≤50 years old; (4) the history of migraine >1 year; (5) with more than 6 times of migraine attacks in the previous 3 months; (6) age between 18 and 65 years old; (7) the patients voluntarily joined this study with informed consent.

### 2.5. Exclusion Criteria

The exclusion criteria were as follows: (1) times of using analgesics for acute headache>10 times per month; (2) alcohol or other drug abuse; (3) primary disease of the liver, kidney, hematopoietic system, cardiovascular system, or cerebrovascular system; (4) psychiatric conditions; (5) hypersensitivity to the trial drug; (6) pregnancy and lactation.

### 2.6. Interventions

The patients in the experimental group would be provided with CXDT-HFG, while the control group would take placebo. Both of them were provided by Hua Run San-Jiu Pharmaceutical Co. LTD. Mix the drugs or placebo with warm-water, and take them twice daily (separately taken in the morning and evening). Both groups were permitted to use the necessary analgesics during an acute migraine attack as well as accepted regular management.

The experimental drug CXDT-HFG is composed of Chuanxiong Rhizoma (Chuanxiong, 12 g), Cyathulae Radix (Chuan Niuxi, 10 g), Dioscoreae Hypoqlaucae Rhizoma (Chuan Bixie, 20 g), Chrysanthemi Flos (Juhua, 6 g), *Uncaria rhynchophylla* Pamuluscum Uncis (Gouteng, 20 g), *Tribuli terrestris* Fructus (Bai Jili, 10 g), Coicis Semen (Yi Yiren, 20 g), Amomi FructusRotundus (Bai Doukou, 6 g), Pinelliae Rhizoma Preparatum (Zhi Banxia, 6 g).

The placebo of single Chinese medicine formula is consisted of dextrin, lactose, caramel pigment, and bitters. Caramel pigment is provided by Shanghai Love Food Industry Company and bitters are manufactured by Zhejiang Deere pharmaceutical factory.

There are better similarities in some ways of dosage, shape, and predominant flavor between the experimental drug CXDT-HFG and placebo.

Permitted medication and specification: generally the patients who have 2 days of headache attacks per week will be permitted to use acute drug treatment; patients should choose the analgesics commonly used without the effect of migraine prevention; the intensity of headache should be measured by VAS and patients should choose corresponding analgesics according to different pain degree. All analgesics used and their doses and effect should be recorded in the diary.

Management standard of participants: (1) patients need to record the headache diary seriously so as to fully understand the time and characteristics of headache attacks. (2) Patients should have a regular lifestyle, the work and rest on schedule, quit smoking, and moderate alcohol consumption. (3) Regulate and refresh emotion and avoid disposition stimulation. (4) Light diet, avoid spicy, and greasy food, reduce the intake of chocolate, coffee, cheese, and other contour tyrosine foods and sausage, ham, hot dogs, and other bacon meats. (5) Patients need to do timely medical examination and treatment if the nature, the degree, and the frequency of headache have changed.

### 2.7. Outcome Measures

The outcome measures included the changes of migraine days, attack frequency, analgesic consumption, and the proportion of responders (defined as the proportion of patients with a reduction of migraine days and times by at least 50% [18, 20]) from baseline to weeks 4, 8, 12 and follow-up period. The frequency of migraine attacks is the main evaluation index, which indicates improvement of the migraine to the maximum extent [22]. Thus the frequency of migraine attacks per 4 weeks should be the primary efficacy measure. The secondary outcome measure was the proportion of responders in attack frequency and migraine days during treatment and follow-up period compared with the baseline period. Inaddition, VAS scores and intensity for pain (mild, moderate, and severe) were assessed at weeks 4, 8, 12 and follow-up period (because VAS scores and pain intensity were not recorded in headache diary retrospectively for nearly 3 months, the 4 weeks' VAS scores and intensity would be seen as baseline at this point). Additionally, patients were required to record adverse events in their headache diaries.

Patients filled in a headache diary on time and recorded the time, frequency, location (the forehead, top, temporal, and back of the head), VAS scores, intensity (mild, moderate, and severe), types of pain, analgesics consumption during migraine acute attacks, and concomitant symptom (nausea, vomiting, photophobia, phonophobia, etc.) in detail, as follows: (1) the days of migraine attacks refer to the onset of the migraine days (provided migraine attack in one day, it will be deemed to one day); (2) a migraine attack that was interrupted either by sleep or treatment but relapsed within 48 hours was required to be documented as a single attack. (3) VAS scores (quantitative index for pain): let the patient himself point out that most representative number of pain; (4) Pain Intensity on the basis of the Numeric Rating Scale (NRS-11) which has been widely used for the assessment of pain [23],was graded according to the number represent pain that patients point out from the scaleplate of VAS: 0 stands for no headache, score range 0~4 stands for mild headache, score range 4~7 stands for moderate headache, score range 7~10 stands for severe headache.

### 2.8. Statistical Methods

Statistical analysis was performed with SPSS (13.0) program for Windows (SPSS, Chicago, IL, USA). Data was managed through the online facility established by the Traditional Chinese Medicine Clinical Foundati**o**n Institute of medicine of the China Academy of the Chinese Medical Sciences, and was analyzed on the full analysis set (FAS), and the per-protocol set (PPS) for adherence. The result of data analysis was mainly for PPS in this trial. The measurement data were expressed as mean ± standard deviation, to check the data of all groups with normal test and homogeneity of variance test. Data analysis was performed by nonparametric statistics to these unmatched normally distributed measurements, including age, course of disease, migraine days, and attacks frequency, etc., which were compared between groups using Mann-Whitney *U* test. Results were shown with 95% confidence interval (95% CI). Measurement data before and after treatment were compared using ANOVA for repeated measures. Headache intensity classification and the proportion of responders were analyzed by *χ* ^2^ test. The level of significance was set at 0.05, if *P* < 0.05, there were statistical differences.

## 3. Results

### 3.1. Dropouts

During the study, 22 patients dropped out, a rate of 14.7% (13 from the CXDT-HFG group 13.1% and 9 from the placebo group 17.6%). Among these, 1 patient was rejected for not meeting inclusion criteria (1 from the CXDT-HFG group), 15 patients dropped out during the treatment period (10 from the CXDT-HFG group, 5 from the placebo group) because of lack of efficacy, pregnancy, etc. And 4 patients dropped out for adverse events (2 from the CXDT-HFG group, 2 from the placebo group). The other 2 patients were lost to followup due to change of contact information (2 from the placebo group). The reasons for the dropouts in the 2 groups are detailed in Figure 1.

### 3.2. Characteristics of Demography and Baseline

The demographic and baseline parameters with the PPS population were shown in Table 1, which showed that the 2 groups were comparable at baseline. The Table 1 summarizes the main baseline characteristics of the 128 patients based on PPS. Participants of CXDT-HFG group had a mean age of 35.77 years and 73.3% (63) were women while the placebo group had a mean age of 34.58 years and 73.8% (31) were women. There were no statistical differences (*P* > 0.05) between the two groups in migraine days, attack frequency, course of disease, consumption of analgesics, all the baseline characteristics of the 2 groups were similar (Table 1).

### 3.3. Days of Migraine Attacks

The baseline of migraine days in the CXDT-HFG group was 4.48 ± 4.12 days and 4.13 ± 4.33 days in the placebo group. At 12 weeks and follow-up period, the migraine days in the CXDT-HFG group decreased to 1.44 days and 1.06 days, respectively, whereas in the placebo group, the migraine days reduced to 1.93 days and 1.69 days, respectively. In other words, the mean reduction of migraine days in the CXDT-HFG group were 3.04 days, compared with 2.20 days in the placebo group at 12 weeks (95% CI, −4.13 to −1.93 versus 95% CI, −3.74 to −0.66; *P* = 0.033), and a mean reduction of 3.42 days in the CXDT-HFG group compared with 2.44 days in the placebo group at follow-up period (95% CI, −4.49 to −2.34 versus 95% CI, −3.97 to −0.90; *P* = 0.042).

**Figure 2:**
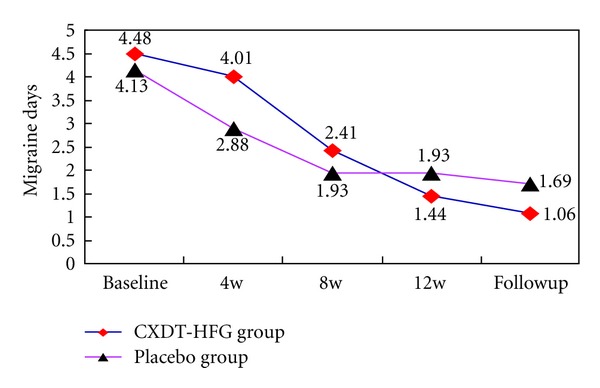
The trend of the changes for migraine days (PPS).

And there was significant difference in the proportion of patients with a reduction of migraine days by at least 50% between the CXDT-HFG group and the placebo group at follow-up period (83.7% versus 64.3%, *P* = 0.014) (Table 1; Figure 2). 

### 3.4. Frequency of Migraine Attacks

**Figure 3:**
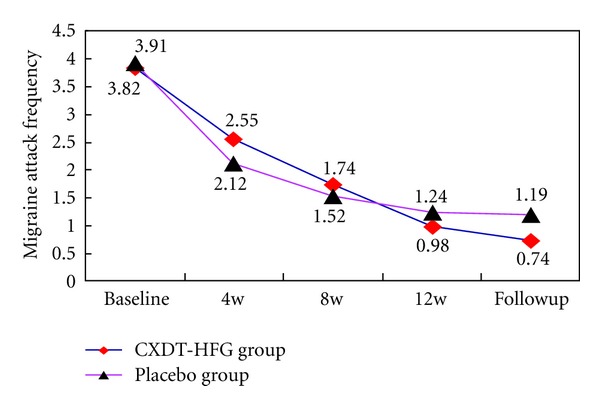
The trend of the changes for migraine attack frequency (PPS).

The baseline of migraine attack frequency in the CXDT-HFG group is 3.82 ± 2.16 times and 3.91 ± 4.32 times in the placebo group. After 12 weeks' treatment, the frequency of migraine attacks of the CXDT-HFG group reduced 2.84 times, while the placebo group reduced 2.67 times (95% CI, −3.35 to −2.33 versus 95% CI, −4.11 to −1.24; *P* = 0.043). At follow-up period the attack frequency of the CXDT-HFG group reduced 3.08 times from baseline and the placebo group reduced 2.72 times (95% CI, −3.57 to −2.58 versus 95% CI, −4.17 to −1.28; *P* = 0.033). However, no significant difference was found in the proportion of patients with a reduction of migraine attack frequency by at least 50% between the CXDT-HFG group and placebo group (Table 1; Figure 3).

### 3.5. VAS Scores and Pain Intensity

The mean VAS scores have significantly decreased in the CXDT-HFG group from 4.96 ± 2.01 at 4 weeks to 1.66 ± 2.15 at follow-up period, whereas in the placebo group from 4.90 ± 2.09 at 4 weeks to 2.92 ± 2.51 at follow-up period (*P* = 0.005). According to VAS scores, the pain degree is divided into four levels with no pain, mild, moderate, and severe. At 12 weeks and follow-up period, there were significant differences between the 2 groups about the pain degree (*P* = 0.037, *P* = 0.013) (Table 2; Figures 5 and 6). 

### 3.6. Acute Medication

**Figure 4:**
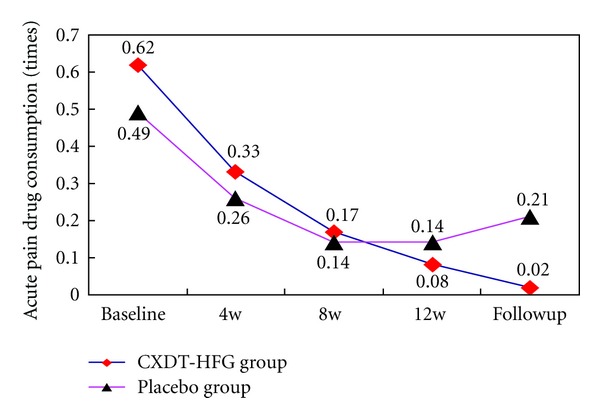
The trend in the change for acute pain drug consumption (PPS).

The number of patients using acute pain drugs, such as aspirin, phenacetin, or ibuprofen, etc., has no significant difference between the 2 groups from baseline to follow-up period (*P* > 0.05) (Table 1; Figure 4).

### 3.7. Safety and Tolerability

A total of 15 (11.72%) subjects in the randomized population experienced mild adverse events during the study period. Treatment-related adverse events were reported in 11.63% of the CXDT-HFG group (10 patients) and in 11.90% of the placebo group (5 patients). The main adverse events were as follows, maculopapula, eyelid edema, palpitations, abdominal pain and nausea, and they were resolved without sequelae after treatment was withdrawn. No clinically significant serious adverse events were reported in subjects on the aspects of liver and renal functions, blood, and urine routines before and after treatment.

## 4. Discussion

### 4.1. Summary of Main Findings

**Figure 5:**
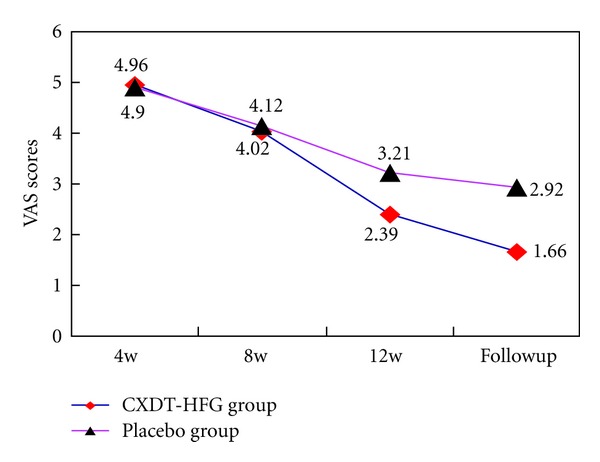
Mean changes of VAS scores between 2 groups (PPS).

**Figure 6:**
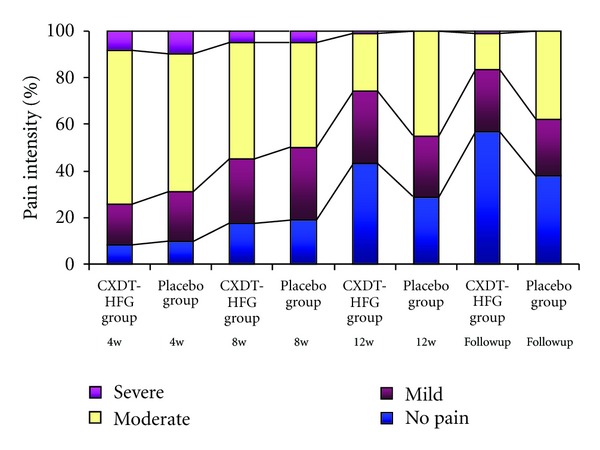
The percentage of pain intensity during the study (PPS).

This trial revealed Traditional Chinese herbal CXDT-HFG to be better than placebo in reducing the days of migraine attacks, frequency, and pain intensity at 12 weeks and follow-up period (*P* < 0.05) as well as in the reduction of VAS scores at follow-up period. And along with the extension of treatment time, the days and frequency of migraine attacks and pain intensity still improved in the CXDT-HFG group. Whereas no significant difference was found between the 2 groups in consumption of acute pain drug (Figures 2, 3, 4, 5, 6).

At the follow-up period, we found that the placebo group presented rebound trend in migraine days and frequency, meanwhile the number of analgesics increased gradually. However the efficacy of the CXDT-HFG group continued to exist, and the decreasing trend was seen in the using of analgesics. 

Traditional Chinese herbal medicine has been practiced in China for thousands of years and vast experience has been accumulated for using medicinal herbs for clinical treatment of diseases. Although the biological mechanism of CXDT-HFG as traditional Chinese herbal medicine in improving the clinical consequence of migraine is not exactly clear, its analgesic effect and reduction of the days of migraine attacks, frequency, and pain intensity play an important role. First, researchers point out herbal medicines have held a place of importance in remedying headaches effectively and safely [17]. Second, according to the theory of TCM, Rhizoma Chuanxiong (Chuanxiong), originates from the plant *Ligusticum* chuanxiong Hort, which is used in TCM to “remove blood stasis and expel wind evil to relieve pain.” And *Uncaria rhynchophylla* (Gouteng) can clear liver heat and flatten rising liver yang as well as Chrysanthemums (Juhua). Modern chemical studies indicate that they have the effect of adjusting nerves, antioxidant, anti-inflammatory, sedation, and analgesic [24–26]. Mechanism research of Chinese herbs for the treatment and prophylaxis of migraine should be further studied.

Overall, the study suggested that CXDT-HFG was efficient in reduction of the frequency of migraine attacks and in alleviating pain degree at week 12, there may be follow-up treatment and prophylaxis effect existing.

### 4.2. Strengths and Limitations of the Study

Migraine is the patient's subjective feeling, so there may be selective bias in grouping as well as measuring bias and hybrid bias in clinical effect assessment. Thus blinded intervention is necessary [27]. This study is a randomized, double-blind, multicenter, placebo-controlled trial, which can reduce bias factors and improve the reliability and scientific validity of the clinical research.

Migraine has the clinical characteristic of relapsing-remitting, and the study mainly chose the remission period for clinical research. Due to the long observation schedule and strict standard of cases inclusion/exclusion, the patients' compliance is poor and future research will be needed to strengthen the management and education of patients to improve the clinical research compliance, and control the rate of dropout.

### 4.3. Implications for Clinical Practice

Migraine is a recurrent disease and its acute episode and remission period have different pathogenesis characteristics and clinical manifestations. The United States migraine evidence-based guidelines [28] point out that the main goal of acute migraine attacks is to relieve the pain quickly and prevent recurrence; the prevention objective of migraine is to reduce the frequency of migraine attacks and relieve pain severity. The advantage of TCM for migraine lies in the preventative treatment during remission period. CXDT-HFG has certain clinical effect in the preventative treatment of migraine sufferers with “the syndrome of Liver Wind and Blood Stasis,” with low adverse reaction. So it is one kind of relatively safe TCM in clinical treatment and prophylaxis for migraine.

### 4.4. Unanswered Questions and Future Research

The application of TCM must base on the theory of syndrome differentiation and treatment. Patients at different states of migraine need different therapeutic methods. In order to make the clinical efficacy of CXDT-HFG used repeatability, we need to expand sample size and do a larger sample, randomized, double-blind, parallel-controlled clinical research, in which we choose an effective drug for migraine which is accepted internationally as the comparison drug to evaluate the clinical effect characteristics of CXDT-HFG for treatment and prevention of migraine further accurately and objectively. 

## 5. Conclusion

CXDT-HFG could reduce the days and frequency of migraine attacks and relieve pain intensity, especially in the prevention of migraine. In addition further research could be conducted on the mechanism of CXDT-HFG for migraine prophylaxis.

## Figures and Tables

**Table 1:** Baseline characteristics of the study participants and primary outcome measures (PPS, *N* = 128).

	CXDT-HFG group (*n* = 86)		Placebo group (*n* = 42)		P* values
	Mean ± SD	95% CI	Mean ± SD	95% CI
Age, years	35.77 ± 11.60	(33.28, 38.25)	34.58 ± 9.85	(31.52, 37.65)	0.738
Sex					
Male, *n* (%)^#^	23 (26.7%)	—	11 (26.2%)	—	0.947
Female, *n* (%)	63 (73.3%)	—	31 (73.8%)	—	—
Course of disease (months)	86.26 ± 88.10	(67.37, 105.14)	82.12 ± 72.76	(59.45, 104.79)	0.716
Migraine days^§^					
Baseline	4.48 ± 4.12	(3.59, 5.36)	4.13 ± 4.33	(2.78, 5.47)	0.572
4 w	4.01 ± 5.11	(2.92, 5.11)	2.88 ± 2.05	(2.24, 3.52)	0.548
Improvement from baseline	−0.46 ± 4.36	(−1.40, 0.47)	−1.25 ± 4.89	(−2.77, 0.28)	0.755
8 w	2.41 ± 3.34	(1.69, 3.12)	1.93 ± 1.52	(1.45, 2.40)	0.848
Improvement from baseline	−2.07 ± 4.42	(−3.01, −1.12)	−2.20 ± 4.77	(−3.68, −0.71)	0.438
12 w	1.44 ± 3.22	(0.75, 3.22)	1.93 ± 2.28	(1.22, 2.64)	0.054
Improvement from baseline	−3.04 ± 5.12	(−4.13, −1.93)	−2.20 ± 4.95	(−3.74, −0.66)	0.033^△^
Follow-up period	1.06 ± 3.13	(0.39, 1.73)	1.69 ± 2.35	(0.96, 2.42)	0.019^‡^
Improvement from baseline	−3.42 ± 5.04	(−4.49, −2.34)	−2.44 ± 4.94	(−3.97, −0.90)	0.042^△^
Migraine attack frequency^§^					
Baseline	3.82 ± 2.16	(3.36, 4.28)	3.91 ± 4.32	(2.57, 5.26)	0.350
4 w	2.55 ± 2.00	(2.12, 2.97)	2.12 ± 1.21	(1.74, 2.50)	0.430
Improvement from baseline	−1.27 ± 1.63	(−1.62, −0.92)	−1.79 ± 4.52	(−3.20, −0.39)	0.641
8 w	1.74 ± 1.57	(1.41, 2.08)	1.52 ± 1.13	(1.17, 1.88)	0.686
Improvement from baseline	−2.07 ± 2.03	(−2.51, −1.64)	−2.39 ± 4.65	(−3.84, −0.94)	0.404
12 w	0.98 ± 1.35	(0.69, 1.27)	1.24 ± 1.08	(0.90, 1.57)	0.085
Improvement from baseline	−2.84 ± 2.36	(−3.35, −2.33)	−2.67 ± 4.60	(−4.11, −1.24)	0.043^△^
Follow-up period	0.74 ± 1.32	(0.46, 1.03)	1.19 ± 1.35	(0.77, 1.61)	0.033^‡^
Improvement from baseline	−3.08 ± 2.32	(−3.57, −2.58)	−2.72 ± 4.63	(−4.17, −1.28)	0.033^△^
Acute pain drug consumption (times)^§^					
Baseline	0.62 ± 1.33	(0.33, 0.91)	0.49 ± 1.10	(0.15, 0.83)	0.506
4 w	0.35 ± 1.19	(0.09, 0.60)	0.26 ± 0.83	(0.00, 0.52)	0.888
8 w	0.17 ± 0.93	(−0.02, 0.38)	0.12 ± 0.45	(−0.02, 0.26)	0.627
12 w	0.08 ± 0.47	(−0.02, 0.19)	0.14 ± 0.57	(−0.03, 0.32)	0.371
Follow-up period	0.02 ± 0.15	(−0.01, 0.06)	0.21 ± 0.75	(−0.02, 0.45)	0.066
Responder rate (migraine days)^#^					
12 w	62 (72.1%)	—	28 (66.7%)	—	0.528
Follow-up period	72 (83.7%)	—	27 (64.3%)	—	0.014^‡^
Responder rate (attack frequency)^#^					
12 w	70 (81.4%)	—	29 (69.0%)	—	0.117
Follow-up period	75 (87.2%)	—	31 (73.8%)	—	0.059

w: weeks.

CI: confidence interval; PPS: per-protocol sets; significant difference, *P* < 0.05. Data presented as mean ± SD, number (percentage) and 95% CI.

*P for comparison with control group.

^#^ *P* values based on Chi-square test.

^§^ *P* values based on repeated measures.

^‡^ *P* < 0.05, for date comparison between groups.

^△^ *P* < 0.05, for *D* value from baseline comparison between groups.

**Table 2:** Changes in VAS scores and pain intensity. (PPS, *N* = 128).

Variable	Group	4 w	8 w	12 w	Follow-up period
	CXDT-HFG group (*n* = 86)	4.96 ± 2.01	4.02 ± 2.33	2.39 ± 2.41	1.66 ± 2.15
VAS scores^§^	Placebo group (*n* = 42)	4.90 ± 2.09	4.12 ± 2.41	3.21 ± 2.45	2.92 ± 2.51
	P* values	0.893	0.679	0.060	0.005^‡^

	CXDT-HFG group (*n* = 86)				
	No pain, *n* (%)	7 (8.14%)	15 (17.44%)	37 (43.02%)	49 (56.98%)
	Mild, *n* (%)	15 (17.44%)	24 (27.91%)	27 (31.40%)	23 (26.74%)
	Moderate, *n* (%)	57 (66.28%)	43 (50.00%)	21 (24.42%)	13 (15.12%)
	Severe, *n* (%)	7 (8.14%)	4 (4.65%)	1 (1.16%)	1 (1.16%)
Pain intensity^#^	Placebo group (*n* = 42)				
	No pain, *n* (%)	4 (9.52%)	8 (19.05%)	12 (28.57%)	16 (38.10%)
	Mild, *n* (%)	9 (21.43%)	13 (30.95%)	11 (26.19%)	10 (23.81%)
	Moderate, *n* (%)	25 (59.52%)	19 (45.24%)	19 (45.24%)	16 (38.10%)
	Severe, *n* (%)	4 (9.52%)	2 (4.76%)	0 (0.00%)	0 (0.00%)
	P* values	0.684	0.673	0.037^‡^	0.013^‡^

W: weeks. Significant difference, *P* < 0.05. Data presented as mean ± SD, number (percentage).

*P for comparison with control group.

^#^ *P* values based on Chi-square test.

^§^ *P* values based on repeated measures.

^‡^ *P* < 0.05. Statistical differences for comparison between groups.
